# LTR-retrotransposon dynamics in common fig (*Ficus carica* L.) genome

**DOI:** 10.1186/s12870-021-02991-x

**Published:** 2021-05-17

**Authors:** Alberto Vangelisti, Samuel Simoni, Gabriele Usai, Maria Ventimiglia, Lucia Natali, Andrea Cavallini, Flavia Mascagni, Tommaso Giordani

**Affiliations:** Dipartimento di Scienze Agrarie, Alimentari e Agro-ambientali, Università di Pisa, Via del Borghetto 80, 56124 Pisa, Italy

**Keywords:** Genome structure and evolution, Long terminal repeat retrotransposons, Plant retrotransposon dynamics, Retrotransposon expression, Retrotransposon insertion time, *Ficus carica* L

## Abstract

**Background:**

Long Terminal Repeat retrotransposons (LTR-REs) are repetitive DNA sequences that constitute a large part of the genome. The improvement of sequencing technologies and sequence assembling strategies has achieved genome sequences with much greater reliability than those of the past, especially in relation to repetitive DNA sequences.

**Results:**

In this study, we analysed the genome of *Ficus carica* L., obtained using third generation sequencing technologies and recently released, to characterise the complete complement of full-length LTR-REs to study their dynamics during fig genome evolution. A total of 1867 full-length elements were identified.

Those belonging to the *Gypsy* superfamily were the most abundant; among these, the *Chromovirus/Tekay* lineage was the most represented. For the *Copia* superfamily, *Ale* was the most abundant lineage. Measuring the estimated insertion time of each element showed that, on average, *Ivana* and *Chromovirus/Tekay* were the youngest lineages of *Copia* and *Gypsy* superfamilies, respectively. Most elements were inactive in transcription, both constitutively and in leaves of plants exposed to an abiotic stress, except for some elements, mostly belonging to the *Copia/Ale* lineage. A relationship between the inactivity of an element and inactivity of genes lying in close proximity to it was established.

**Conclusions:**

The data reported in this study provide one of the first sets of information on the genomic dynamics related to LTR-REs in a plant species with highly reliable genome sequence. Fig LTR-REs are highly heterogeneous in abundance and estimated insertion time, and only a few elements are transcriptionally active. In general, the data suggested a direct relationship between estimated insertion time and abundance of an element and an inverse relationship between insertion time (or abundance) and transcription, at least for *Copia* LTR-REs.

**Supplementary Information:**

The online version contains supplementary material available at 10.1186/s12870-021-02991-x.

## Background

The fig tree (*Ficus carica* L.) belongs to the Moraceae family and is a deciduous fruit tree considered amongst the oldest domesticated tree species [1]. During the last decades the interest in fig considerably increased because of both the nutraceutical properties of the fruit and economic value of this species [2, 3]. *F. carica* cultivation is spread throughout the Mediterranean area, Middle East, and Asia. These regions are affected by a high temperate climate during the summer, which increases salinity in the soil due to reduced water availability [4]. The fig tree has showed moderate resistance to saline stress compared to other fruit trees [5, 6]. Recently, the *F. carica* genome has been completely sequenced [1], showing a total of ~ 333 Mbp arranged in 13 pseudo-chromosomes, containing 37,840 genes, mostly composed of repeated sequences of which long terminal repeat retrotransposons (LTR-REs) constitute the vast majority [1]. However, a comprehensive analysis of fig LTR-REs is still lacking.

Retrotransposons (REs) are usually widespread in all eukaryotes and constitute a large part of the genome, especially in plant species [7]. They can move across chromosomes, thus, multiplying their number into the genome of host plants. In fact, REs replicate by a ‘copy and paste’ mechanism, exploiting the expression of an RNA intermediate that is converted into cDNA before being inserted back into a different chromosomal location. The most abundant order of REs in plant species shows two long terminal repeats (LTRs) flanking a polyprotein region, containing encoding domains necessary to replicate the sequence, such as protease, reverse transcriptase, RNAse H, and an integrase, with an additional GAG domain that encodes for virus-like particles protein [7, 8]. The majority of plant LTR-REs are represented by the two main superfamilies, *Copia* and *Gypsy*, which differ principally in sequence divergence and the organization of polyprotein domains. Based on sequence similarities, these elements can be distinguished into several lineages [7, 9, 10].

The RE component of a plant genome is subject to turnover [11, 12]; in fact, REs may increase their number in a relatively short time. However, REs may also be removed from the genome through unequal homologous and illegitimate recombination [13, 14].

Retrotransposons play an important role in the evolution of a species because their mobility produces genetic variation [15]. They can favour chromosome rearrangements through illegitimate recombination [13] between sites lying far in the same chromosome or in different chromosomes [16]. Moreover, REs may insert within or near a gene, altering its coding sequence or its splicing pattern [17]. Perhaps more importantly, the insertion or removal of a RE in proximity of a gene may change its transcription regulation, altering its expression rate in response to different stimuli [18–21].

Because of the potentially harmful effects of their mobility, REs are generally epigenetically inactivated by the host, for example, through DNA methylation [22, 23]. Consequently, the insertion of a RE in a locus may lead to modification of the epigenetic setting of that insertion site [17]. As a matter of fact, REs are known to regulate the epigenetic setting of the genome and chromatin organisation and structure [17].

Nevertheless, LTR-REs can elude silencing machinery and move into new sites, leading to potential genetic changes. For example, the activation of LTR-REs has been reported in many plant species; in many cases, the regulation of RE expression was shown to depend on external stimuli, such as those related to biotic and abiotic stresses [24–27].

In genome sequences obtained using high throughput second-generation sequencing technologies which use relatively short DNA sequences to be assembled, the assembly of LTR-REs can be complicated by the fact that such REs are repeated (in many cases, highly repeated) in the genome and are by far longer than a single read. Furthermore, in some cases, the assembled sequence may be composed of sequence reads derived from multiple copies of the repetitive element collapsed together, resulting in a consensus rather than in a real sequence.

Third-generation sequencing (TGS) technologies [28], that produce very long sequence reads, have allowed the production of high-quality genome assemblies [29–31], overcoming many of the problems related to short-read sequencing assemblies, such as the resolution of repetitive sequences [22, 32].

Recently, a high-quality genome sequence of *Ficus carica* cv. Dottato [1] was produced using single-molecule, real-time (SMRT) sequencing developed by Pacific Biosciences (PacBio, Menlo Park, CA, USA), the first attempt to sequence the genome of this heterozygous species by long-read assembly, thus providing a high-quality genomic resource. In fact, PacBio technology can successfully resolve the complex repetitive fraction of most genomes, such as those of plants, by spanning complete repetitive regions with unique sequences. The availability of such a contiguous reference genome assembly is a crucial prerequisite for the correct identification of repeats by a de novo approach.

In the previous work [1], repeated sequences were identified and subjected to a preliminary characterization. The repeated sequences were used to mask the assembly, thus obtaining the count of the global amount of bases associated to putative repeated regions, but no analysis were performed on single full-length elements. Here, we present a comprehensive study of fig LTR-REs, with the identification and complete characterisation of all full-length elements. In particular, after identification and structural characterisation, we analysed the abundance and estimated insertion time of each isolated element in the genome of *F. carica*. Furthermore, we used a transcriptomic approach to analyse the expression of LTR-REs during abiotic stress, i.e., in fig leaves from plants of the same cultivar (Dottato) exposed to short and prolonged periods of saline stress [33]. In fact, LTR-RE expression represents the first step of retrotransposon activation, with possible consequences on the genetic structure of a species. Finally, the data on abundance, age, and transcription activity of all LTR-REs were related to provide a global view on the dynamics of REs in a precise plant system.

## Results

### Full-length LTR-REs isolation and structural characterisation

A structural analysis allowed us to identify a total of 1867 unique full-length long terminal repeat retrotransposons (LTR-REs).

A comparison between the two available fig genome assembly, the one produced using third generation sequencing technology [1] and the previous NGS-based assembly [34] showed that the number of retrieved full-length elements was by far higher using long-read sequencing. In fact, only 20 full-length LTR-REs versus 1867 could be retrieved from the NGS-based assembly. The total amount of 1887 LTR-REs were clustered using 90% sequence similarity thresholds. Only sixteen out of 1867 LTR-REs clustered with elements of NGS-based assembly, further showing the large improvement in repeat assembly obtained using third generation sequencing technology.

Overall, 1010 out of 1867 LTR-REs showed all functional domains, whereas the remaining 857 elements lacked one or more functional domains, hence they can be considered as transpositionally non-autonomous.

**Fig. 1:**
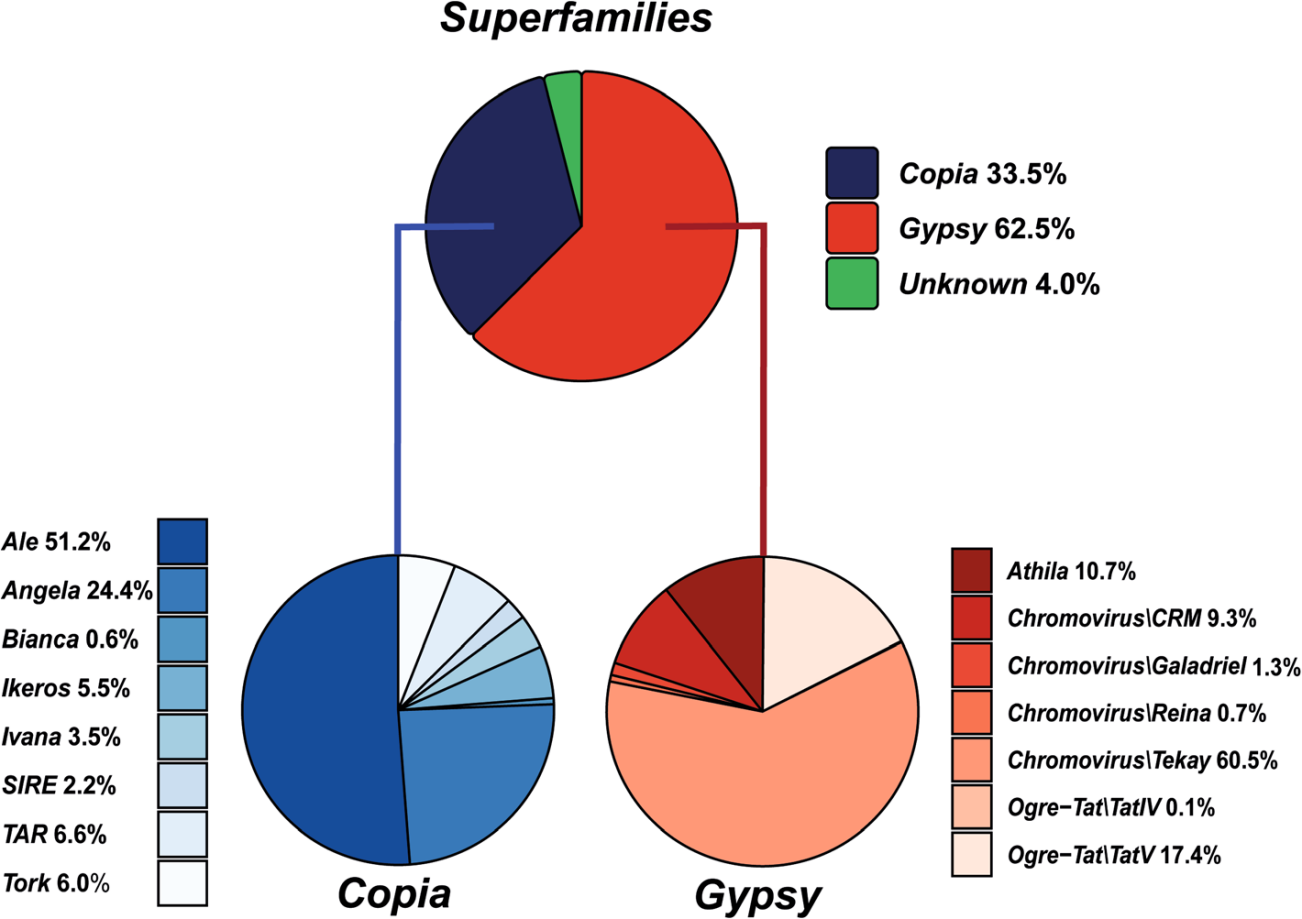
Pie charts of the distribution of full-length LTR-REs in *F. carica* genome considering both superfamilies and *Copia* and *Gypsy* lineages

We were able to annotate 1163 *Gypsy* elements (62.52%) and 623 *Copia* elements (33.49%), i.e., the number of *Gypsy* full-length elements was almost two-fold higher than the *Copia* ones. A total of 74 full-length LTR-REs (3.97%) remained unknown (Fig. 1).

With regards to the *Copia* superfamily, 319 *Ale* (51.20%), 152 *Angela* (24.39%), 4 *Bianca* (0.64%), 34 *Ikeros* (5.45%), 22 *Ivana* (3.53%), 14 *Sire* (2.24%), 41 *TAR* (6.58%), and 37 *Tork* (5.93%) elements were characterised (Fig. 1). The *Ale* lineage was by far predominant, followed by *Angela*. The other identified lineages were underrepresented.

As for the *Gypsy* superfamily, *Chromovirus* elements were the most abundant, being 835 out of 1163 (71.77%), subdivided into 108 CRM (9.28%), 15 *Galadriel* (1.28%), 8 *Reina* (0.68%), and 704 *Tekay* (60.53%) elements. The other *Gypsy* lineages, *Athila* and *Ogre-Tat* were represented by 125 (10.74%) and 203 (17.44%) elements, respectively (Fig. 1).

**Fig. 2:**
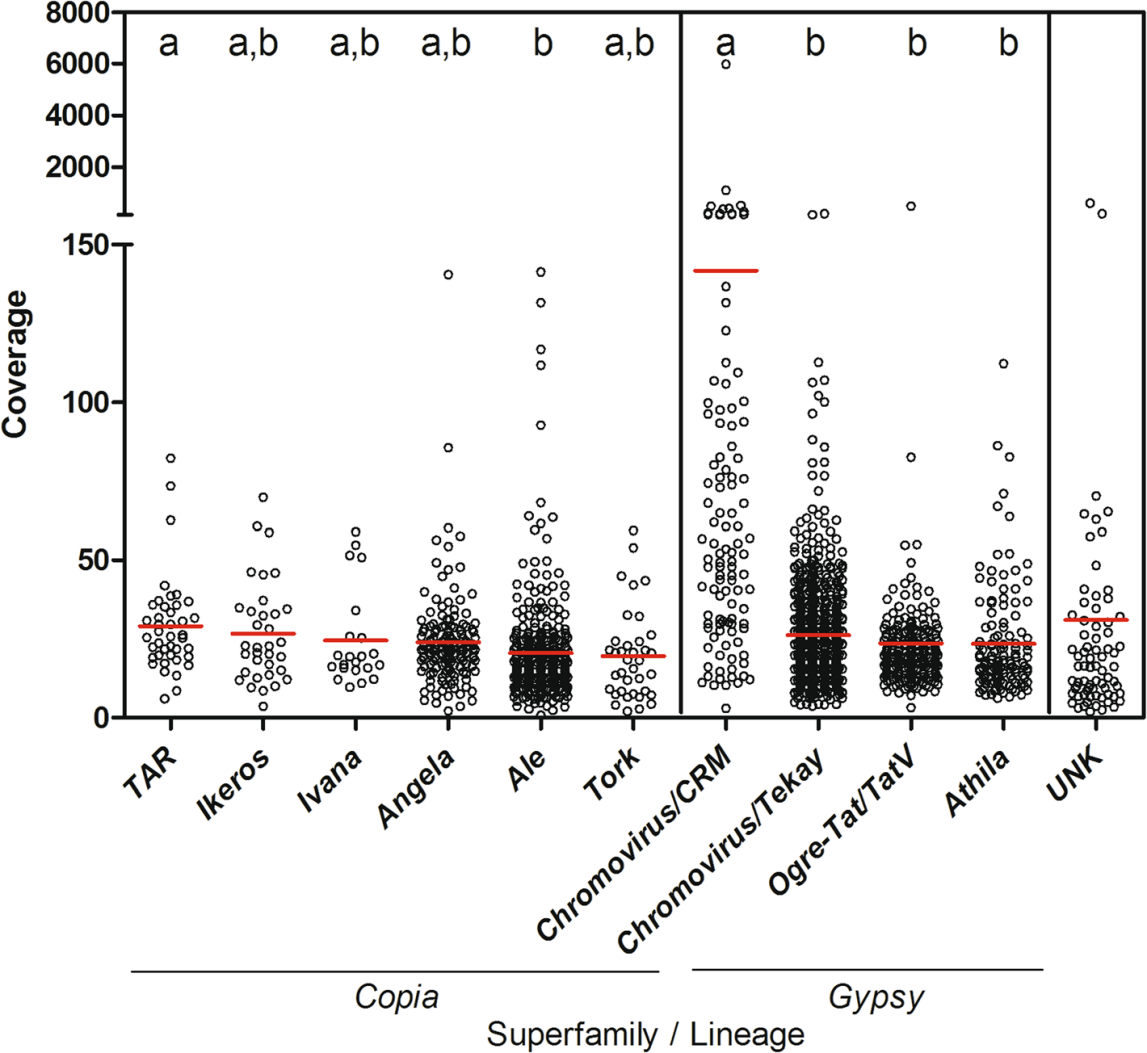
Genomic abundance (showed as average coverage) of full-length LTR-REs for *Copia* and *Gypsy* lineages in the *F. carica* genome. Red bars represent the average value for each lineage. Significant differences (calculated by ANOVA) for each separate group of measurements are indicated by letters a and b: groups sharing the same letter are not significantly different (*p* < 0.05) according to Tukey’s test. UNK = unknown superfamily

Concerning the genomic abundance of LTR-retrotransposons in the *F. carica* genome, mapping Illumina DNA reads to the full-length LTR-retrotransposons (see Methods) showed that *Gypsy* was the most plentiful superfamily. In particular, the most abundant lineage was *Chromovirus/CRM*, with an average coverage of 141.76, followed by *Chromovirus/Tekay*, *Ogre-Tat/TatV,* and *Athila*, showing average coverage of 26.23, 23.58, and 23.47, respectively (Fig. 2). The most represented lineages belonging to the *Copia* superfamily were *TAR*, with an average coverage of 29.06, followed by *Ikeros* (26.75 average coverage), *Ivana* (24.53), *Angela* (23.95), *Ale* (20.49), and *Tork* (19.59) (Fig. 2). Finally, LTR-retrotransposons that were not classified and named as ‘unknown’, showed an overall average coverage of 31.04 (Fig. 2).

### Insertion time of LTR-retrotransposons in the *F. carica* genome

The insertion age of an LTR-RE can be estimated by counting nucleotide substitutions between the LTRs of that same element, which should be identical immediately after the retrotransposition event and then accumulate mutations over time [35]. In our measurements, we applied the *Populus trichocarpa* synonymous substitution rate of 2.36 × 10^− 9^ (see Methods). Analyses were performed separately on the two LTR-RE superfamilies and on the most abundant *Gypsy* and *Copia* lineages.

**Fig. 3:**
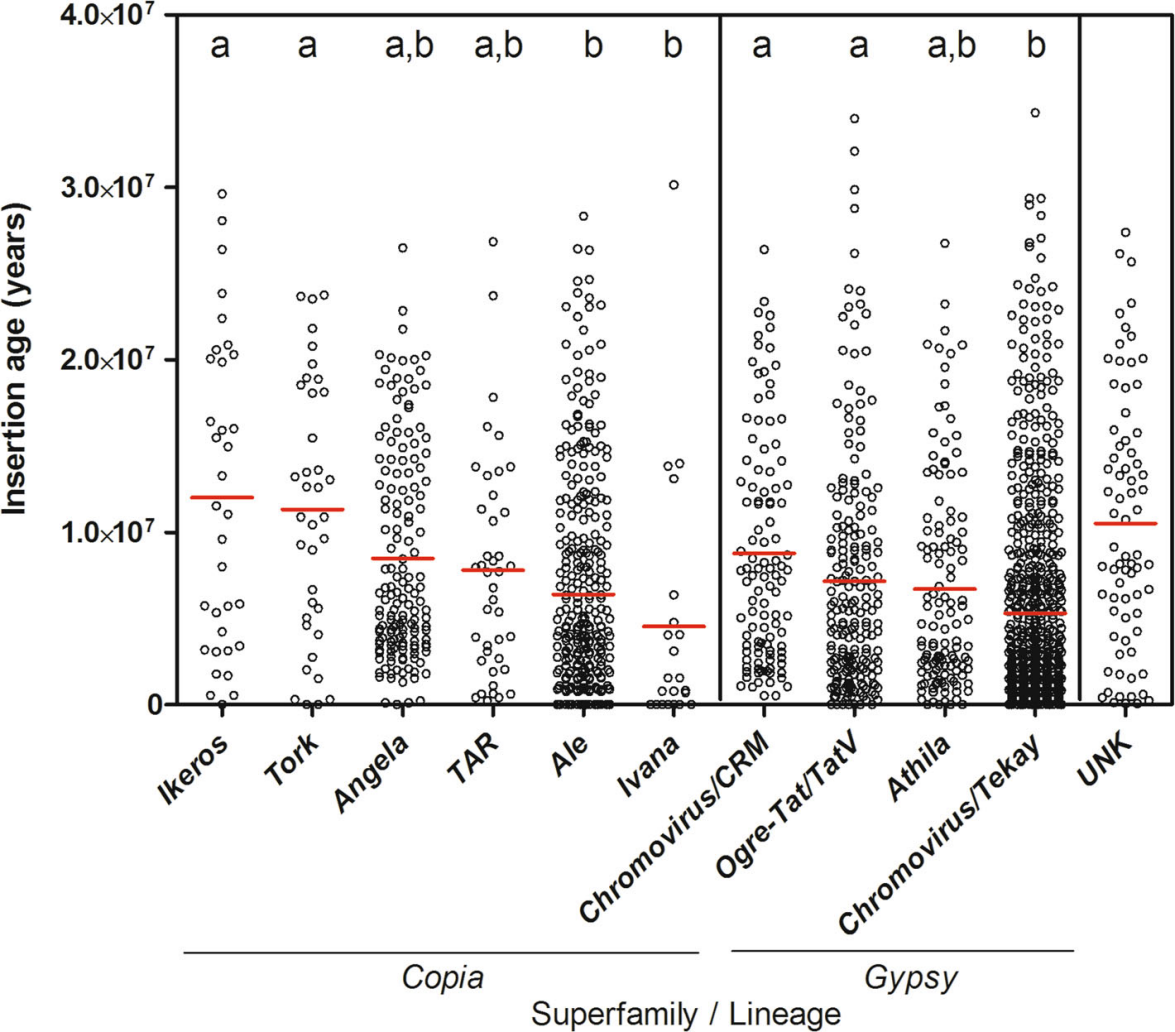
Distribution of *Copia* and *Gypsy* full-length LTR-REs in the *F. carica* genome by insertion age. Red bars represent the average value for each lineage. Significant differences (calculated by ANOVA) for each separate group of measurements are indicated by letters a and b: groups sharing the same letter are not significantly different (*p* < 0.05) according to Tukey’s test. UNK = unknown superfamily

The estimated insertion time of each fig full-length LTR-RE is reported in Fig. 3. On average, older elements belonged to *Copia* (7,9 MY on average) compared to *Gypsy* (6,1 MY). The oldest lineage belonging to the *Copia* superfamily was *Ikeros* (12,0 MY on average), while the youngest LTR-REs belonged to the *Ivana* (4,5 MY) and *Ale* lineages (6,4 MY). The oldest *Gypsy* lineage was *Chromovirus/CRM* (8,8 MY on average), whereas the youngest one was represented by *Chromovirus/Tekay* (5,3 MY). Unknown LTR-REs showed an average estimated insertion time of about 12,5 MY (Fig. 3).

### Expression analysis of LTR-retrotransposons in *F. carica*

The Illumina reads of cDNA from leaves of the control and salt stressed plants of *F. carica* at two time points, 24 and 48 days after the beginning of the experiment, were mapped on 1867 full-length LTR-REs. Overall, a total of 250,464 reads were aligned on the reference set of LTR-REs. On average, 0.22% of reads per library mapped on the sequence set, ranging from 0.16 to 0.31%, depending on the library (Additional file 1).

**Fig. 4:**
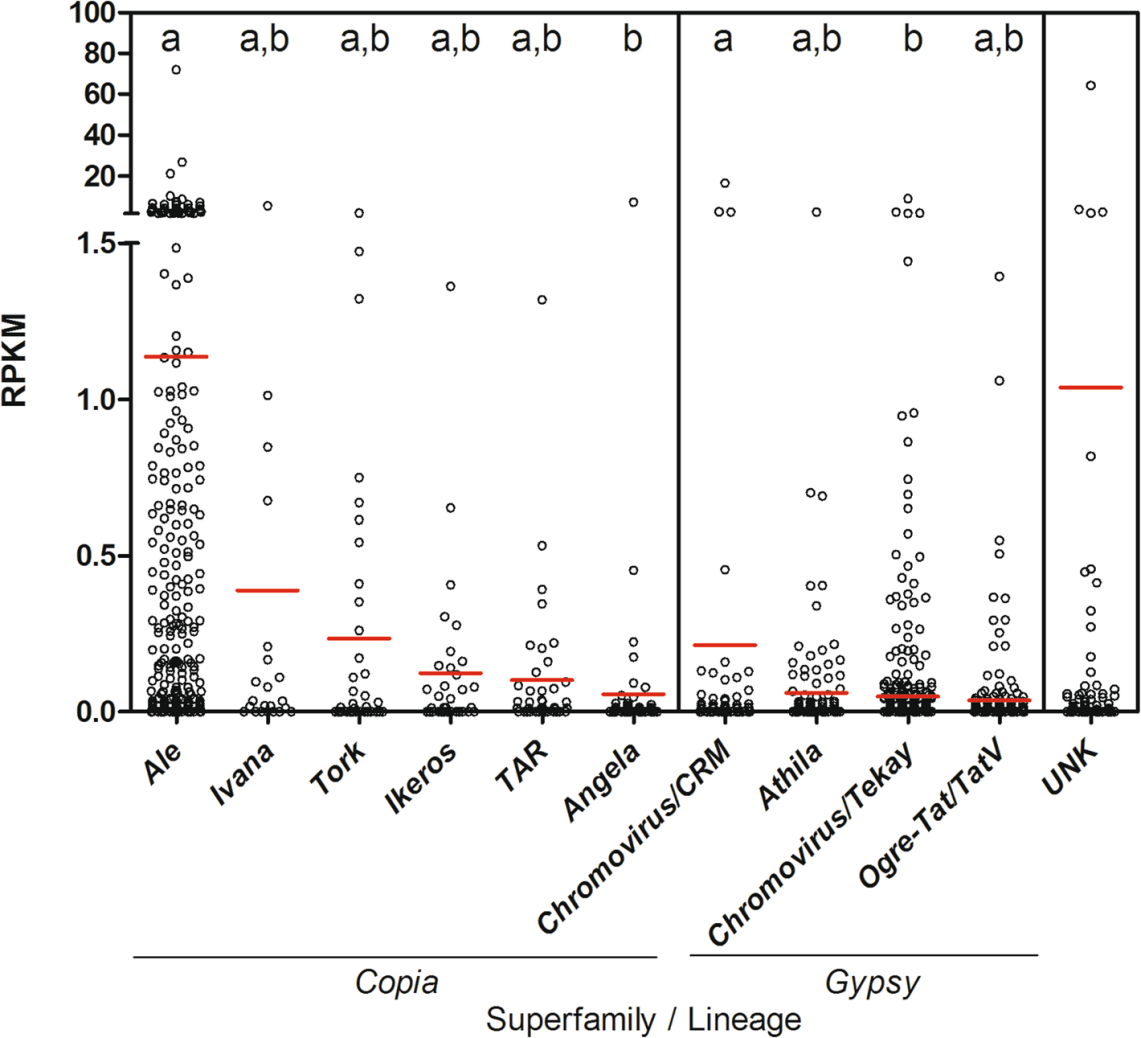
Full-length LTR-REs RPKM distribution for each lineage in the *F. carica* genome. Red bars represent the average value for each lineage. Significant differences (calculated by ANOVA) for each separate group of measurements are indicated by letters a and b: groups sharing the same letter are not significantly different (*p* < 0.05) according to Tukey’s test. UNK = unknown superfamily

Globally, low expression of full-length LTRs was detected for both *Copia* and *Gypsy* superfamilies with an average RPKM of 0.64 and 0.06, respectively (Fig. 4). High percentages of unique reads (i.e., reads mapping to one LTR-RE only) were observed, spanning from 91.8 to 95.6%. Such high percentages of unique reads should ensure high reliability to measured LTR-REs expression values.

*Copia* lineages showed slightly higher expression compared to *Gypsy* ones. The most expressed *Copia* lineage was *Ale* (mean RPKM = 1.13), followed by *Ivana* (0.38) and *Tork* (0.23) (Fig. 4). The most expressed *Gypsy* lineages were *Chromovirus/CRM* (0.21), *Athila* (0.06), and *Chromovirus/Tekay* (0.04).

Considering unknown full-length LTR-REs, we retrieved an average RPKM of 1.04 (Fig. 4). In particular, one UNK element showed a very high RPKM. Further analysis on this element revealed the presence of two putative gene sequences within the unknown element, that might explain the high expression value of this LTR-RE.

We set RPKM > 1, in at least one library, as a threshold to consider a full-length element as expressed during the experiments; based on this threshold, there were 153 expressed LTR-REs out of 1867. Considering the 153 expressed elements, 97 encoded all protein domains and therefore can be considered as intact and autonomous. Fifty-one expressed elements lacked one or more domains and 5 elements resulted to be devoid of domains.

**Fig. 5:**
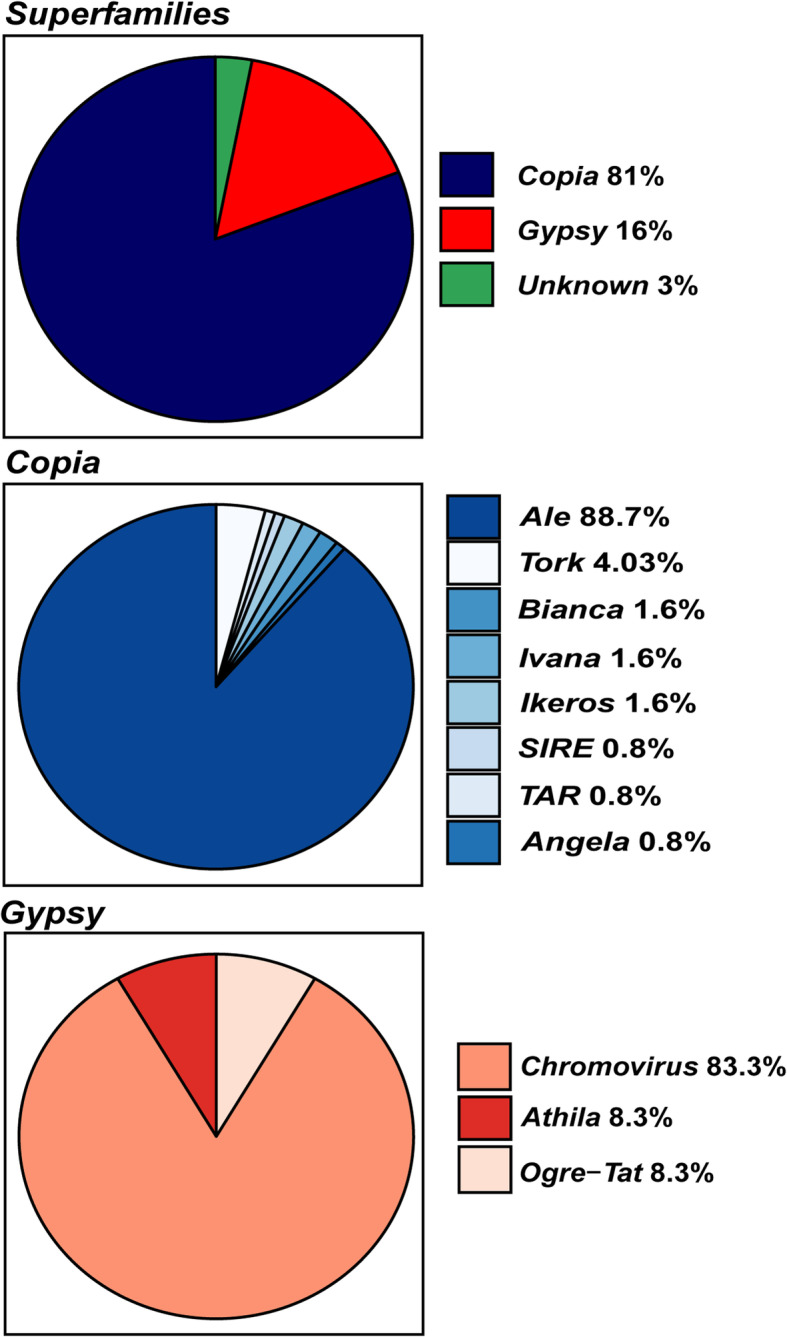
Pie charts of expressed fig LTR-Res, considering both superfamilies and *Copia* and *Gypsy* lineage distributions

The main fraction of expressed LTR-REs belonged to the *Copia* superfamily (81.0%), followed by *Gypsy* (16.0%) and unknown elements (3.0%) (Fig. 5). Concerning lineages, the most represented *Copia* lineages were *Ale* (88.7%) and *Tork* (4.0%), whereas the most represented *Gypsy* lineage was *Chromovirus* (88.3%), followed by *Athila* (8.3%), as shown in Fig. 5. In particular, the expressed *Chromovirus* elements belonged to the four sublineages *Tekay* (60%), *Reina* (20%), *CRM* (15%) and *Galadriel* (5%).

### LTR-retrotransposons transcription analysis in *F. carica* leaves of salt exposed plants

**Fig. 6:**
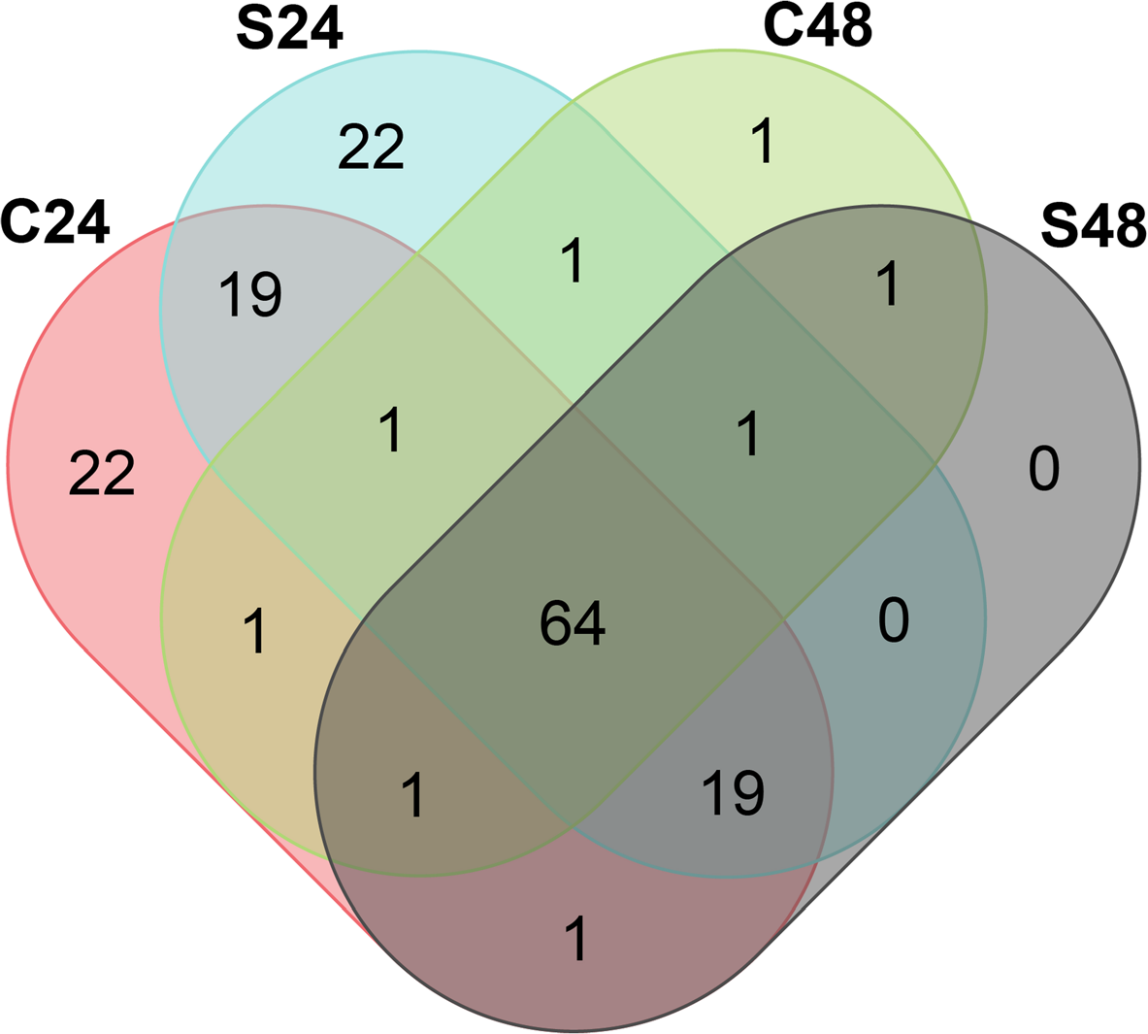
Venn diagram of expressed LTR-REs after 24 and 48 days of salt treatment. C = leaves from control plants, S = leaves from salt treated plants

A total of 153 expressed LTR-REs (i.e., showing RPKM > 1 in at least one RNA-seq library) during the saline stress in *F. carica* leaves were further investigated. Most of these REs (64) were expressed amongst all treatments, as shown in Fig. 6. Nevertheless, we found 22 LTR-REs uniquely expressed in the control and salt stressed leaves of *F. carica* after 24 days of saline treatment and one LTR-RE that was activated in control leaves after 48 days from the beginning of experiment (Fig. 6). We did not find any LTR-REs specifically expressed in the leaves of plants exposed to salt for 48 days.

Concerning the LTR-REs expression level we detected an average RPKM value of 2.74 amongst all libraries with a maximum of 89.56, values similar to low/medium-expressed reference genes that showed a stable expression both in control and salt stressed leaves (Additional File 2). Overall, we detected slightly higher expression during the first time point (24 days after beginning of experiment) for leaves of both control and stressed plants of *F. carica* (Additional File 3), although such differences proved to be not significant by ANOVA statistical analysis.

**Fig. 7:**
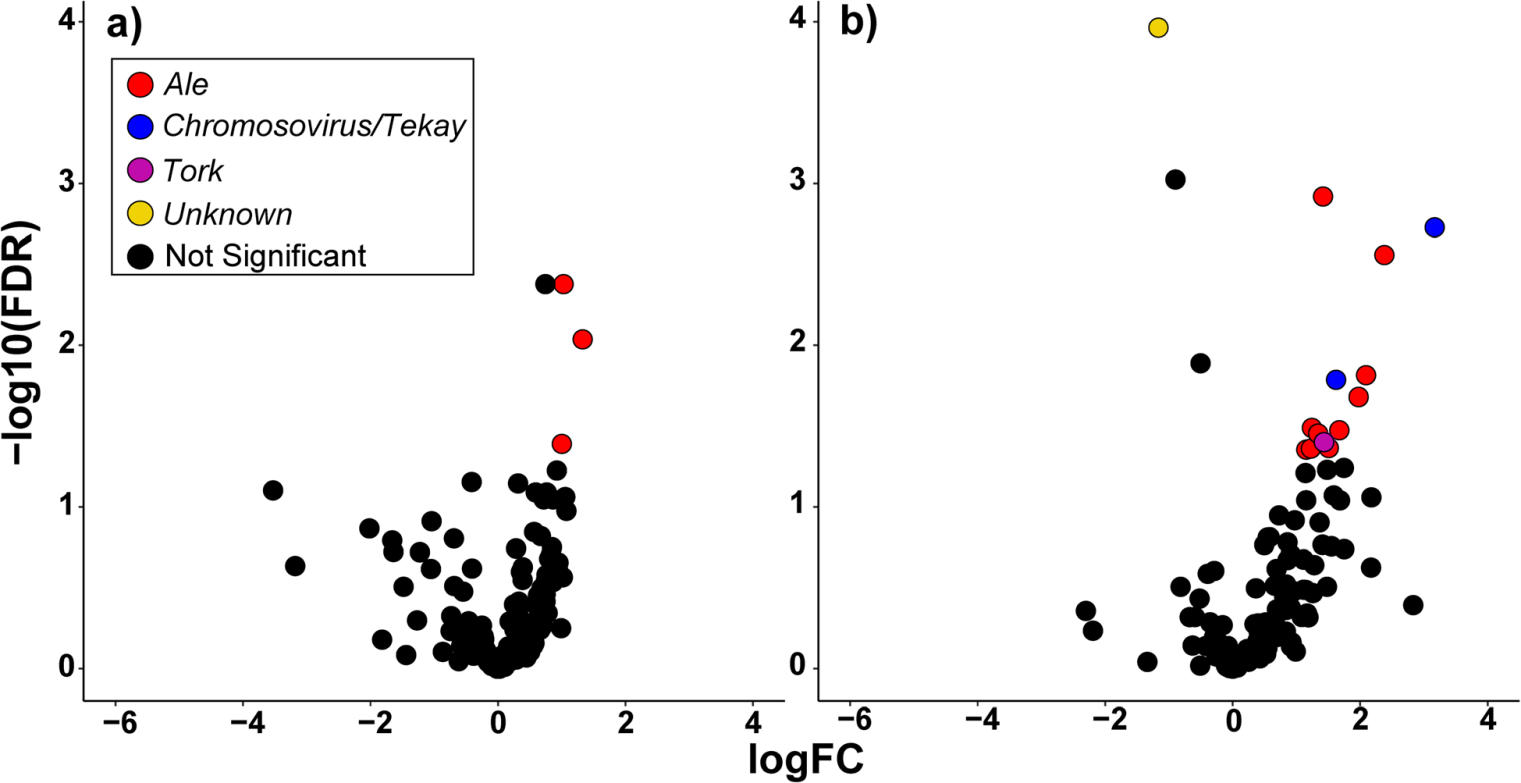
Volcano plots of expressed fig LTR-REs after 24 (**a**) and 48 (**b**) days of salt treatment. Coloured dots represent the differentially expressed LTR-REs. The colour indicates the lineage of the differentially expressed element. Black dots represent elements whose expression was statistically unaffected by salt treatment. FC = fold change, FDR = False Discovery Rate corrected *p*-values

To find differentially expressed LTR-REs during salt stress in fig leaves, we performed a pairwise test between the leaves of control and salt-treated plants at 24 and 48 days of treatment. We detected three *Ale* elements that were overexpressed after 24 days since the beginning of the experiment (Fig. 7a). At the second time point, we retrieved 13 activated LTR-REs (Fig. 7b), belonging to the *Ale* (10 elements), *Chromovirus/Tekay* (2)*,* and *Tork* (1) lineages*.* Finally, one element whose superfamily was not identified was underregulated after 48 days of salt treatment (Fig. 7b).

### Expression of genes lying in close proximity to expressed full-length LTR-retrotransposons

**Fig. 8:**
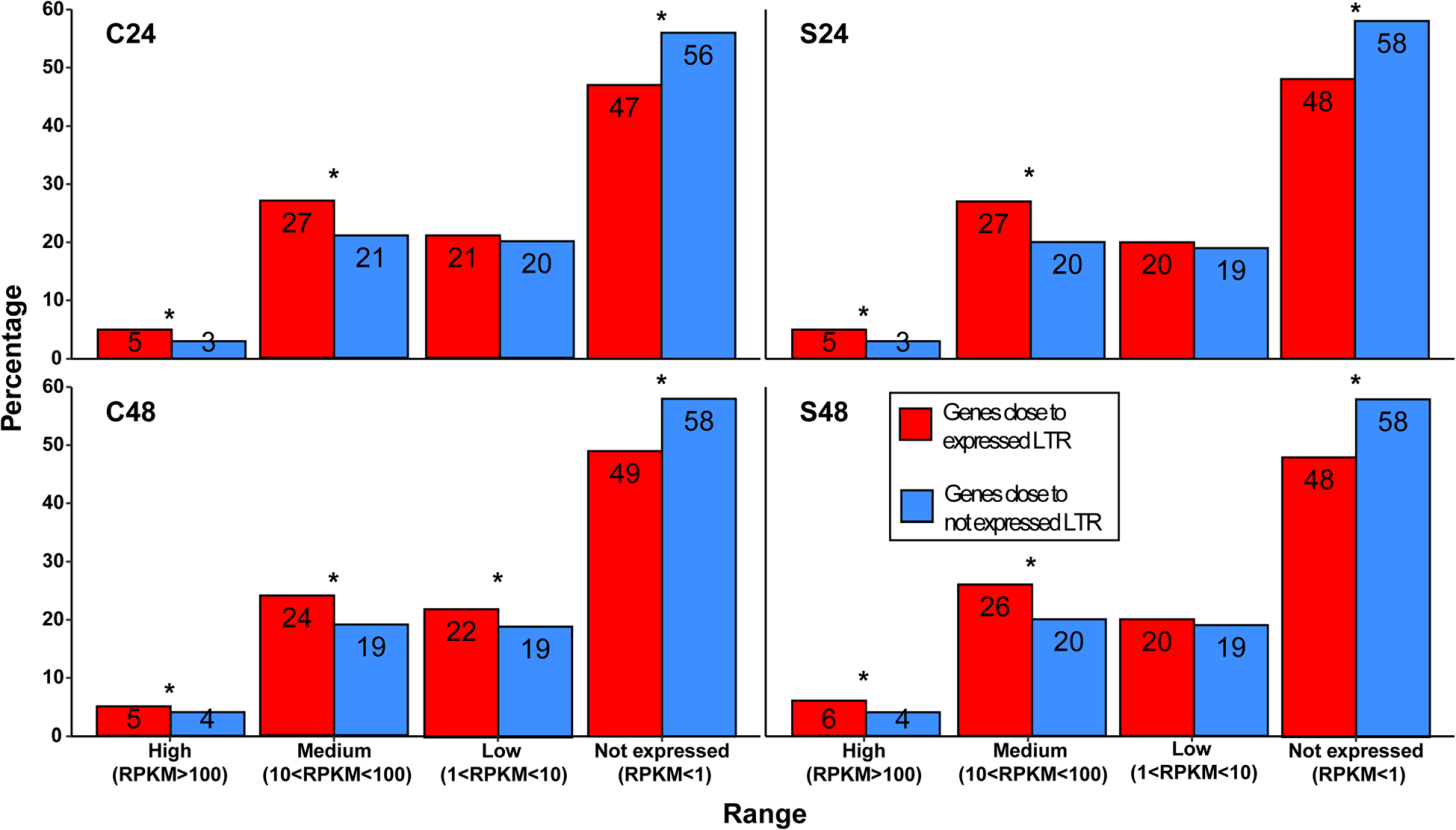
Bar plot of expression pattern for genes lying in close proximity to expressed and not expressed fig LTR-REs. RPKM values were subdivided in categories. Asterisks outline categories that are significantly different (Fisher’s exact test FDR < 0.05) between genes lying close to expressed and to not expressed LTR-REs

The expression and function of genes in the frame of 50 kbp up- and downstream to LTR-REs expressed (153 elements) or not expressed (1714) during salt stress in fig leaves were investigated. To analyse expression patterns, we separated genes in four categories: not expressed (RPKM < 1), lowly expressed (1 < RPKM < 10), medium expressed (10 < RPKM < 100), and highly expressed (RPKM > 100). Overall we detected 1816 genes lying in close proximity to expressed LTR-REs. Of these, not expressed genes were the most abundant (48% average), followed by medium (26% average), lowly (21% average), and highly expressed (5% average) genes (Fig. 8). A comparison of expression level between genes lying in close proximity to expressed or not expressed LTR-REs showed a significantly higher percentage of highly and medium expressed genes lying close to active LTR-REs than to inactive ones in leaves of both the control and salt treated plants (Fig. 8).

**Fig. 9:**
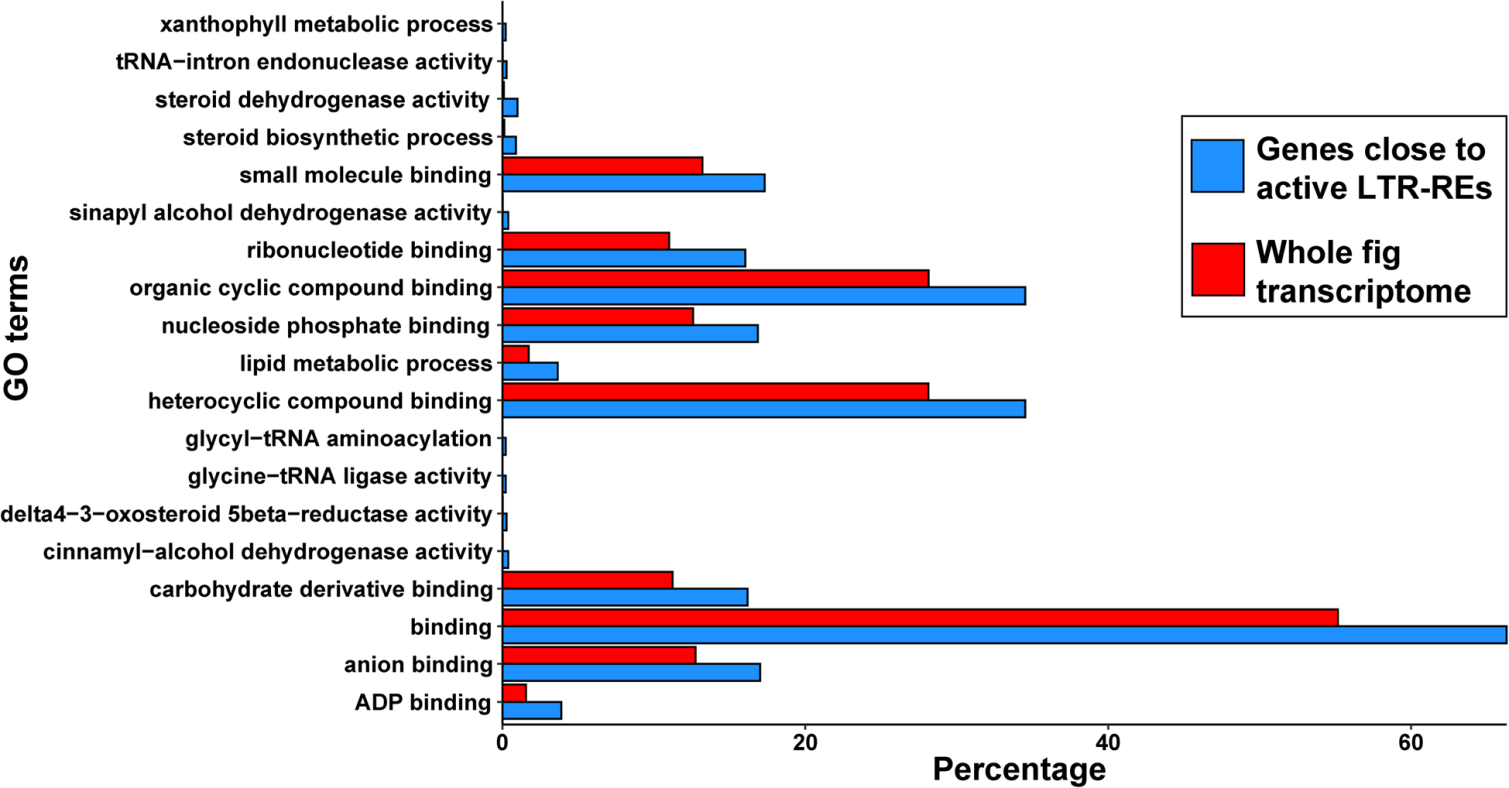
Enrichment analysis for Gene Ontology (GO) terms of genes close to expressed LTR-REs summarised by REVIGO. GO terms were compared to the whole *F. carica* transcriptome; only significantly enriched GO terms were reported

In order to investigate the occurrence of specific GO-terms in genes close to active LTR-REs, we used an enrichment analysis. This analysis compared the GO-terms in genes proximal to LTR-REs against the GO-terms of the whole *F. carica* transcriptome, by using a Fisher’s exact Test. Only GO terms showing a FDR corrected *P*-Value < 0.05 were considered. A total of 19 GO terms were specifically enriched compared to the whole *F. carica* transcriptome (Fig. 9). Among these, we found terms involved into lipid metabolic process, ribonucleotide binding, and steroid metabolic processes.

### LTR-retrotransposons dynamics in *F. carica* genome

The relationships between expression, genome abundance, and estimated insertion time of full-length LTR-REs were analysed. Such correlations were not significant, probably because LTR-REs in a genome are a very heterogeneous population, made especially of lowly repeated and inactive elements. However, the measured regression lines can highlight trends in the relationships between LTR-RE abundance, insertion age and expression.

**Fig. 10:**
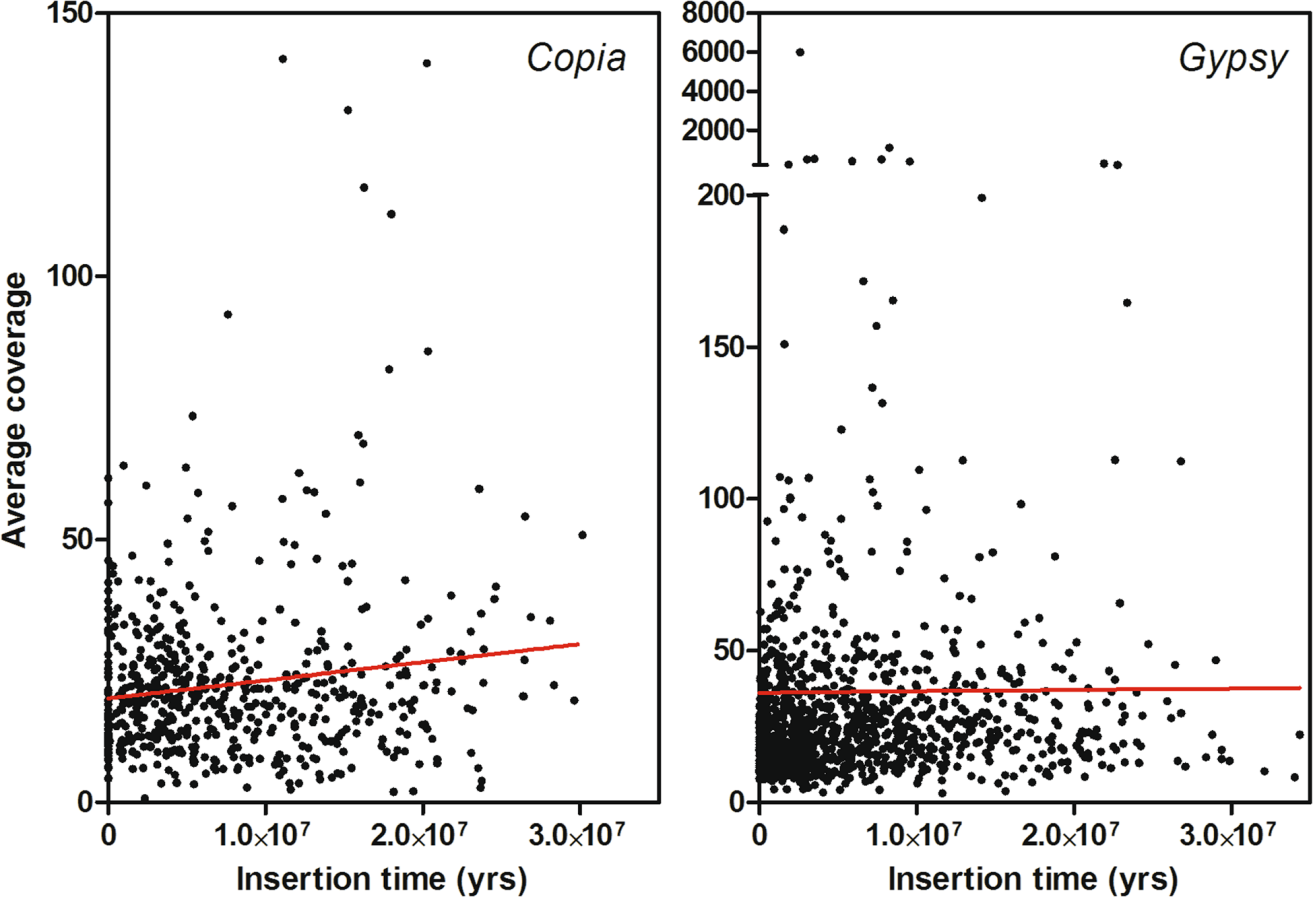
Correlation between genomic abundance (reported as average coverage) and estimated insertion time (in years) for *Copia* and *Gypsy* full-length LTR-REs in the *F. carica* genome

For example, the correlation between estimated insertion time and genome abundance (measured by average coverage) of *Copia* and *Gypsy* superfamilies was not significant; nevertheless, the regression lines suggested that older *Copia* LTR-REs were more abundant than younger ones in the *F. carica* genome, whereas *Gypsy* LTR-REs genome abundance was not different between young and old elements (Fig. 10).

**Fig. 11:**
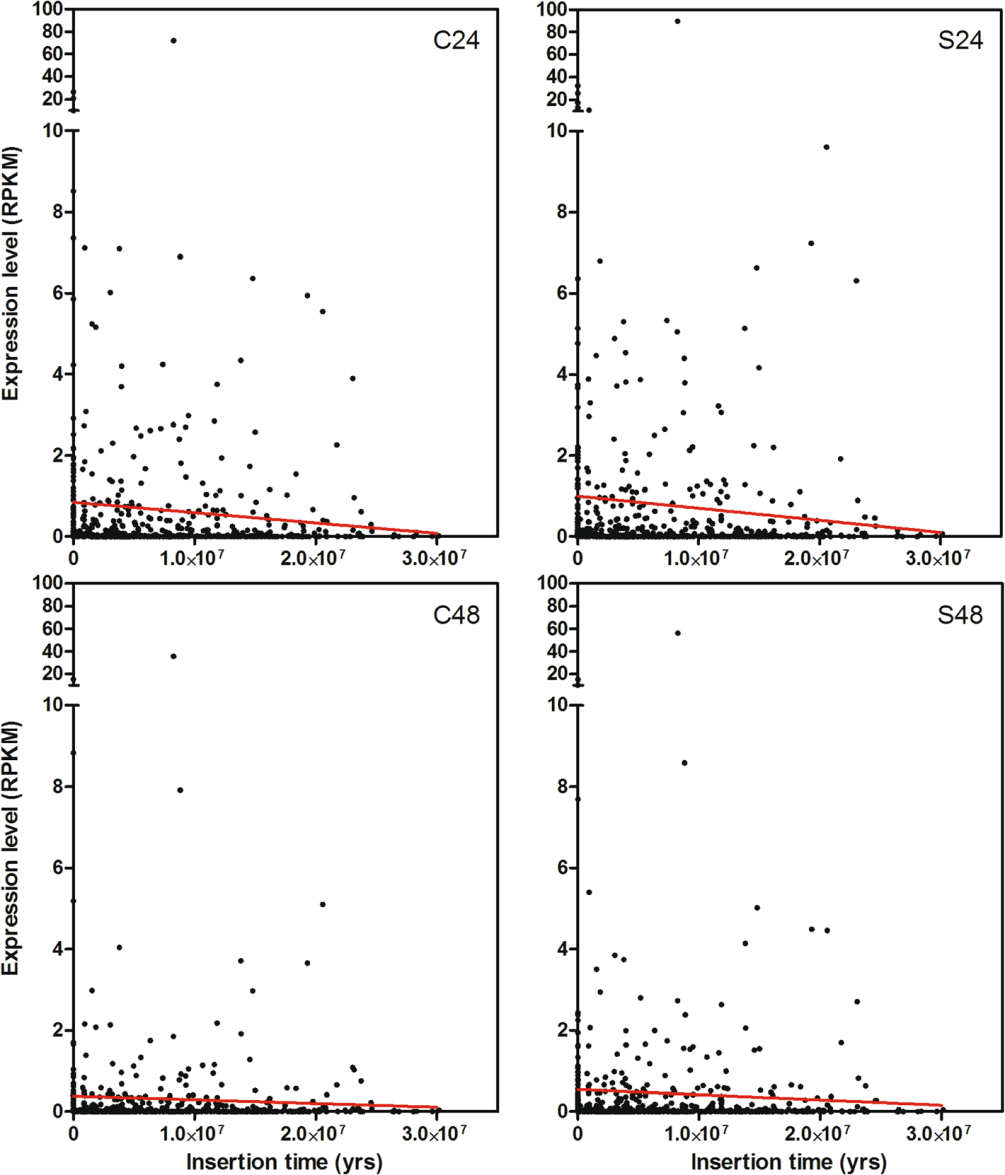
Correlation between expression values, measured as RPKM mean and estimated insertion time (years) for *Copia* full-length LTR-REs in the *F. carica* genome

Regarding the relationship between measured insertion time and expression level, LTR-REs belonging to the *Gypsy* superfamily were not analysed because they all showed very low RPKM values. The regression lines between age and expression level of full-length LTR-REs belonging to the *Copia* superfamily, in leaves of both control and salt-treated plants, showed that expression values tended to decrease with age (Fig. 11), although the correlation was not significant.

**Fig. 12:**
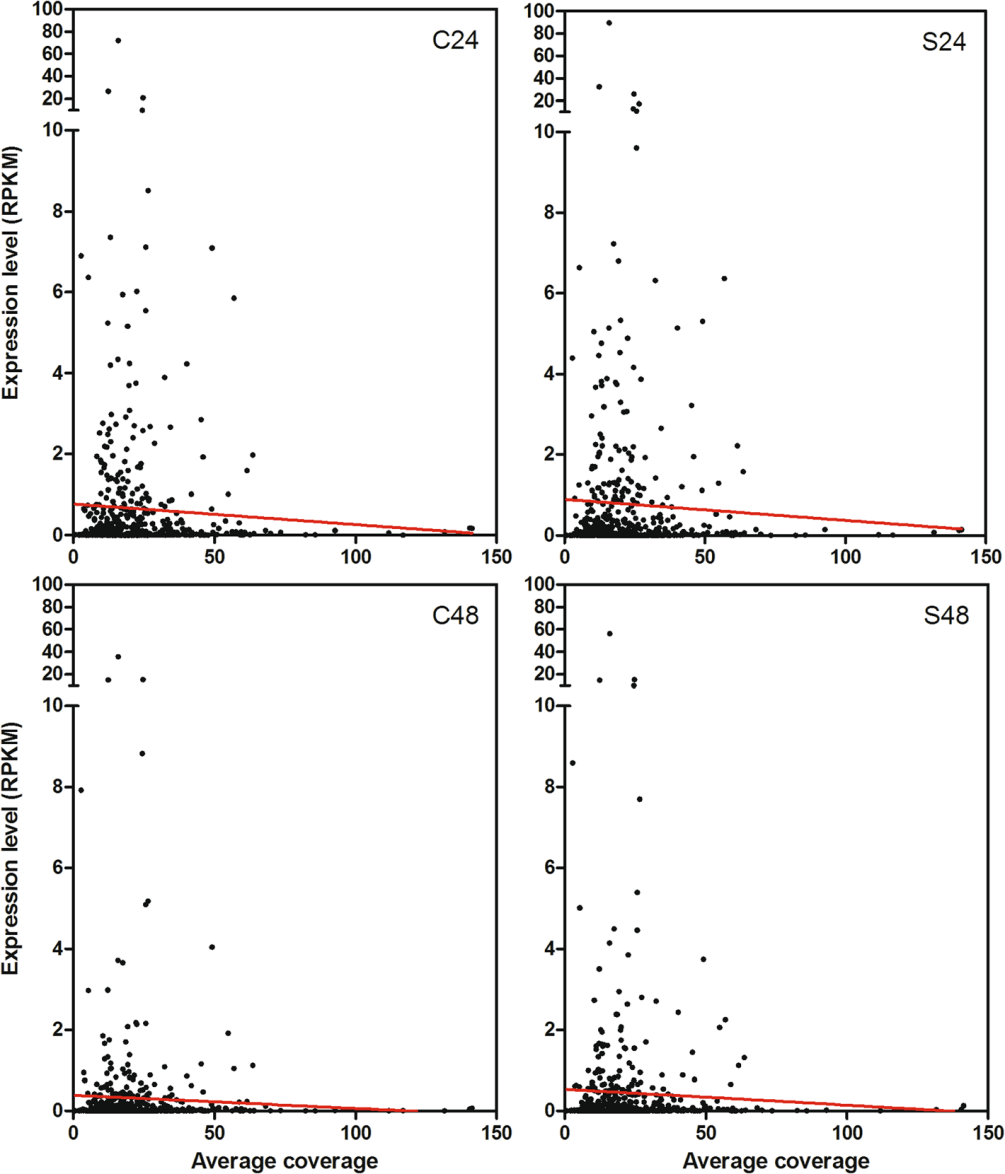
Correlation between expression level (reported as RPKM mean) and genome abundance (reported as average coverage) for *Copia* full-length LTR-REs in the *F. carica* genome

Finally, we analysed the correlation between genomic abundance (average coverage) and expression values (RPKM mean) of *Copia* full-length LTR-REs (*Gypsy* elements were not analysed because they were very lowly expressed). Although there was no statistical significance also in this correlation, regression lines outlined a trend according to which the more a LTR-RE was expressed, the less abundant (lowest average coverage) it was in the genome, in all treatments (Fig. 12).

In addition, by comparing the slopes of regression lines of the interaction estimated insertion time/expression level between control and salt-treated samples, we found that this value increased between control and salt-stressed leaves both after 24 and 48 days since the beginning of the experiment (Table 1), suggesting possible activation of older LTR-REs during abiotic stress. Similarly, regression lines of genomic abundance against expression level showed an increase of the slopes during salt treatment after 24 and 48 days compared to control leaves. These data indicate the possible activation of most abundant full-length LTR-REs in the *F. carica* genome during saline treatment.

**Table 1:** Regression line slopes for each control (24 d and 48 d, C24 and C48) and salt (24 d and 48 d, S24 and S48) treatment in the correlations of expression level vs. estimated insertion time or vs. genome abundance of fig full-length LTR-REs

Correlation	Treatment	Slope
Expression level / Insertion time	C24	−10,000
	S24	− 78.395
	C48	− 129.900
	S48	−90.595
Expression level / Genome abundance	C24	−0.11
	S24	−0.075
	C48	−0.25
	S48	−0.15

## Discussion

The aim of our work was to provide complete characterisation of the LTR-REs of a species, *F. carica*, and their dynamics in the genome, through the study of all full-length elements identifiable in the genome of this species. Analyses of the repeatome have been generally conducted using low coverage sequencing and clustering analyses (see for example, Mascagni et al. [36] and Zagorski et al. [37]) and rarely consider full-length elements, for example, in *Populus trichocarpa* [9] and *Potentilla micrantha* [10]. Nevertheless, the improvement of DNA sequencing and sequence assembling strategies that allow the use of long sequence reads are now achieving genome sequences with much greater completeness than before, especially in relation to the repetitive component of the genome. Therefore, genomes sequenced using these new procedures allow for a vastly more precise and reliable characterisation of repeated elements.

In the case described in this study, we analysed the genome of *F. carica*, obtained by combining PacBio and Illumina sequencing and recently released [1]. This species has a relatively small genome (356 Mbp [38]), and TEs represent roughly one third of the genome [1], a proportion similar to that found in other small-sized genomes [39] and higher than that measured on the previously available fig genome sequences (16% [34]). This difference is most likely due to missing or collapsed repeated sequences, a typical phenomenon widely observed in assemblies generated through NGS technologies, especially when assemblies are highly fragmented. Other studies have already highlighted how long-read technologies can successfully improve the resolution of repetitive genomic regions. For example, Oxford Nanopore Technology sequencing of Arabidopsis enabled the resolution of a quantitative trait loci (QTL) previously recalcitrant to BAC sequencing due to its repeat structure that required reads greater than 20 kb [40]. Other similar observations were highlighted in wheat [41] and maize [42].

In this study, we identified 1867 full-length elements that were classified in relation to their superfamily and lineage. The vast majority of these elements were specific to the PacBio-based assembly, confirming the higher quality of third generation sequencing technology based assemblies to resolve the complex repeated regions of a genome.

The full-length elements of the *Gypsy* superfamily were much more frequent than *Copia*, similarly to other species, such as poplar [9]. At the lineage level, for the *Copia* superfamily, *Ale* elements were the most frequent, similar to other species, such as *Erythrostemon hughesii* [43]. For the *Gypsy* superfamily, the *Chromovirus* lineage (in particular, the *Tekay* sublineage) showed the highest abundance as already reported in other species, like pepper and *Hieracium* [37, 44].

For each full-length element we measured the genome abundance, putative age of insertion, and transcription activity in leaves of control plants and of plants subjected to salt stress [33].

The *Chromovirus* lineage (sublineage *CRM*) showed the highest mean average coverage. *Chromovirus/CRM* elements are mainly located in the centromeres and in the pericentromeric regions, where they probably play a structural role [45–49]. Notably, the full-length elements of this sublineage were about 6% of all full-length elements identified in this work, while their mean average coverage (141.76) was much higher than those of all other lineages (ranging from 30.50 to 19.59), indicating the occurrence, in the fig genome, of many *Chromovirus/CRM* remnants and fragments (i.e., incomplete elements) not recognisable by the software that predicts only full-length LTR-REs, and probably related to the insertion of new elements within other elements (the so-called nested retrotransposons), structures that have already been described in the centromeric regions of other species [50].

Analysis of the insertion age of the full-length elements shows an extreme variability within each lineage, indicating wide insertion time spans, with large overlaps of activity for different elements, as already reported in poplar, sunflower, and mulberry [9, 51, 52]. On average, among the *Copia* elements, the lineages *Ikeros* and *Tork* were significantly older than lineages *Ale* and *Ivana*. Among *Gypsy* LTR-REs, the average age of the *Chromovirus/CRM* and *Ogre* elements was significantly higher than the *Chromovirus/Tekay* elements.

The expression of full-length elements was investigated by mapping Illumina cDNA reads obtained from leaves of plants subjected to salt stress and of control plants on the elements. In the case of repeated sequences, this type of analysis is biased because it is not certain that a cDNA read that maps to a full-length LTR-RE was not actually produced by an RNA transcribed from another element (or from an incomplete element) of the same family, which therefore shares its sequence with the full-length element and is located at another locus. However, the very high percentages of unique reads that mapped on the full-length LTR-REs (from 91.8 to 95.6%) allowed us to establish reliable trends in the expression patterns of each element.

Our results showed that the expression of LTR-REs is generally very low, in both control and salt-exposed plants. This finding was expected because LTR-REs are commonly subjected to strict control that prevents their expression through different mechanisms. One major mechanism for transposon repression is RNA interference, which is mediated by small RNAs originating from double-stranded RNAs produced by the host, which trigger retrotransposon silencing through DNA methylation, chromatin remodelling, and post-transcriptional degradation of RE transcripts [53–55].

Considering RPKM > 1 in at least one library as a threshold for defining a given expressed element, only 153 of 1867 elements were considered expressed in control and salt treated plants. Although *Gypsy* elements represented the vast majority of LTR-RE complements in the fig genome, most expressed elements belonged to the *Copia* superfamily. Qiu and Ungerer [56] found similar results in three wild species of the *Helianthus* genus, in which the most expressed LTR-REs belonged to the *Copia* superfamily and especially to barely repeated families. As a matter of fact, *Gypsy* elements are known to insert especially into heterochromatin [57–59], while Copia LTR-REs tend to insert also into euchromatin regions [58, 60], that are more commonly subjected to transcription.

The presence of RE transcripts in plant tissues have been described in many species, without an apparent induction stimulus or following exposure to environmental changes [61]. The first case has been reported in species like *Citrus sinensis*, sunflower, rice, and poplar [62–66]. In the second group, induction of LTR-RE transcription has been shown when mimicking abiotic and biotic stress [67–74]. Notably, the occurrence of transcripts complementary to a LTR-RE sequence can be considered as i) the first step of LTR-RE activation, which may lead to final retrotransposition or ii) it may represent the production of LTR-RE RNAs to be used for RE silencing.

Concerning the expression of different LTR-RE lineages, the vast majority (88.7%) of the expressed *Copia* elements belonged to the lineage *Ale*, and in the *Gypsy* superfamily, one lineage (*Chromovirus*) accounted for 83.3% of expressed LTR-REs.

In general, salt stress showed little effect on the expression of LTR-REs. During early salt treatment, only three elements (all the *Copia/Ale* lineage) were upregulated. *Ale* elements were also the most represented among those (in total 13 elements) showing significant upregulation after 48 days of exposure to salt. Notably, the elements upregulated after 24 days were not the same as those upregulated after 48 days, suggesting a different type of induction by salt stress for elements of the same lineage.

Using RNA-seq data of leaves of salt-exposed and control plants, after 48 days, we analysed the effects of the presence of a LTR-RE on the expression of genes located in proximity to it (within 50 kbp up- and downstream to each full-length LTR-RE). We observed that most of the genes close to LTR-REs were not expressed. However, the expression of genes close to a LTR-RE was greater when the LTR-RE was expressed than when it was not expressed. This data suggests a reciprocal influence between transposons and genes regarding transcription when they are close together on the chromosome. Such influence is probably linked to epigenetic control (for example, through DNA methylation) of the chromosomal locus, in which genes and LTR-REs are in proximity to each other. Methylation spreading from plant retrotransposons into flanking DNA regions has been reported [75, 76].

It is possible that the insertion of a retrotransposon could imply epigenetic control of expression in its adjacent regions, with consequent repression of transcription along that chromosomal locus [75]. In particular, it was shown that transposable element insertion can modify gene expression pattern even in the frame of thousands base pairs upstream, such as in the case the *teosinte branched1* gene of maize, whose expression pattern was modified by the insertion of a *hopscotch* retrotrasposon at a distance of more than 60,000 bp from the gene [77].

However, it is also possible to hypothesise that, in the case of retrotransposon insertion in the proximity of genes whose expression is necessary to the cell and should be maintained, it may make it more difficult to silence that locus by DNA methylation. In this case, other LTR-RE silencing mechanisms should operate, probably on a post-transcriptional level [78].

By analysing the genes that are located close to expressed LTR-REs, we observed that some GOs are significantly more frequent in genes occurring in regions where expressed elements are found, indicating that the chromosomal regions where these genes are found are probably more difficult to be silenced by DNA methylation. Obviously, other studies are needed, especially in other species, to assess whether there is a relationship of this type between retrotransposons and certain functional categories of genes.

We then tried to evaluate possible correlations between genome abundance, insertion age, and expression of LTR-REs. The resulting regression lines were not significant, probably due to the extreme variability among LTR-REs regarding the insertion age and abundance. Such variability is related to the fact that each LTR-RE can be autonomous in replication in the host genome. Concerning the expression, only a few elements were expressed and the level of expression was generally very low, making it difficult to establish significant correlations. However, the regression lines obtained by our data allowed us to establish trends in LTR-RE dynamics.

Concerning the correlation between genome abundance and insertion age, for the *Copia* superfamily, the oldest elements were also more abundant indicating that, over time, there was continuous production of new copies of each element or that ancient LTR-REs were subjected to a replication burst. In contrast, for the *Gypsy* superfamily, it can be hypothesised that there is stronger control over the replication of the oldest elements, so that their abundance was not greater than that of the youngest ones. Cases have been reported in which even single elements were found to have had bursts of replication activity, such as to increase their genomic abundance in a short time span, in a range of species that include *Vicia pannonica* [79], cotton [80], *Oryza australiensis* [81], and *Helianthus agrestis* [66].

For the correlation between expression and age of insertion, it was possible to evaluate only the *Copia* elements, as the expression of the *Gypsy* elements was too small to allow a correlation study. This is another indication of the greater control that the host puts in place on this superfamily of elements than on the *Copia* superfamily.

Considering *Copia* elements, the expression tended to decrease as the insertion age increased, probably because the host had more time to set up epigenetic control of that element.

The same trend was observed for the correlation between the expression of LTR-REs and their genomic abundance. As a matter of fact, such a relationship was ascertained in several studies, in which the more an element was abundant the more it was recognised and subjected to silencing [15, 65, 82, 83].

## Conclusions

In conclusion, our data provide a complete characterisation of the full-length LTR-REs of *F. carica*, yielding interesting information on the genomic dynamics related to these elements and on their role in gene regulation. The extension of this type of analysis to other species whose genome has been sequenced using third-generation sequencing technologies will allow, through comparative analyses, a better understanding of the importance of these elements in the evolution of species at the genomic level.

## Methods

### Full-length LTR-REs isolation and characterisation

Class I full-length LTR-REs were identified in the highly contiguous fig genome sequence produced by Usai et al. [1] using the PacBio SMRT sequencing technology in combination with Illumina sequencing.

The elements were identified using LTRharvest v1.5.10 [84] and annotated using LTRdigest v1.5.10 [85] and the DANTE tool v1.0.0 provided on the RepeatExplorer Galaxy-based website (https://repeatexplorer-elixir.cerit-sc.cz/galaxy/). LTRharvest was run on the fig genome assembly with the following parameters: -minlenltr 100, −maxlenltr 6000, −mindistltr 1500, −maxdistltr 25,000, −mintsd 5, −maxtsd 5, −similar 85, −vic 10.

The available NGS-based fig genome sequence [34] was also subjected to the same structural-based identification process and a sequence comparison was performed by using CD-Hit est. with similarity threshold set to 90% [86].

tRNA sequences of *Arabidopsis thaliana*, *Populus trichocarpa*, *Vitis vinifera,* and *Zea mays* were retrieved from GtRNAdb (http://gtrnadb.ucsc.edu) for the identification of the primer binding site regions of the identified elements through LTRdigest.

The library of full-length LTR-REs was submitted to the DANTE tool for domain-based annotation. The process of annotation was run with default parameters using REXdb for transposable element protein domains [48] and a BLOSUM80 scoring matrix. The results were filtered by the significance of the protein matches with default parameters. Final annotation was manually checked to remove any nested cases.

In further characterisation of full-length elements, the insertion date of each LTR-RE was estimated through a pairwise sequence divergence comparison of the 5′- and 3′-LTRs. LTR pairwise alignments and distance matrices were calculated using stretcher and distmat tools of the EMBOSS v6.6.0.0 suite, respectively [87], using the Kimura two-parameter model of sequence evolution [88]. The element insertion times were estimated using a mutation rate of 2.36 × 10^− 9^, i.e., two-fold the rate calculated for synonymous substitutions in gene sequences in *Populus trichocarpa* [89], because LTR-REs accumulate more mutations with time compared to gene sequences.

In some cases, the occurrence of protein encoding domains within an element was checked by using Augustus [90] and DANTE under default parameters.

### LTR-REs redundancy analysis

DNA libraries of *F. carica* cv. Dottato were downloaded from the NCBI database (NCBI, Washington, USA, https://www.ncbi.nlm.nih.gov/sra) using accession number SRP109082. The construction of libraries was described by Solorzano Zambrano et al. [91]. DNA sequencing was performed with two Illumina sequencers: MiSeq and HiSeq2000, yielding 125 bp reads. The reads were trimmed using Trimmomatic v0.33 [92] with the following criteria: HEADCROP:19, CROP:100, SLIDINGWINDOW:4:20, MINLEN:100. Illumina adapters were also removed.

High-quality reads were cleaned from organellar sequences by mapping them to the *F. carica* chloroplast and *Morus notabilis* mitochondrial genomes using CLC Genomics Workbench v9.5.3 (CLC-BIO, Aarhus, Denmark) with stringent parameters: mismatch cost = 1, deletion cost = 1, insertion cost = 1, similarity fraction = 0.9, and length fraction = 0.9. The resulting un-mapped reads were collected.

The genomic abundance of LTR-REs in *F. carica* genome was assessed by aligning Illumina DNA reads to the full-length LTR-REs isolated from the assembly [1]. Mapping of Illumina DNA reads to full-length LTR-REs was performed using the CLC Genomics Workbench with the following criteria: mismatch cost = 1, deletion cost = 1, insertion cost = 1, similarity fraction = 0.9, and length fraction = 0.9.

### RNA isolation

Two-year old *F. carica* (cv. Dottato), micropropagated plants were grown in 5 L plastic pots filled with a mixture of 6.4% clay, 8.6% silt, and 85% sand and subjected to salinity stress [33]. Plants were treated with salinity concentrations of 0 and 100 mM NaCl, and two time points corresponding to 24 and 48 days after the beginning of the experiment were chosen. Hence, for each time point, one fully expanded leaf from three plants of the control (0 mM NaCl) and three salt-stressed plants (100 mM NaCl) were collected.

Total RNA was extracted from leaves according to Giordani et al. [65] and genomic DNA contamination removed by DNAse I (Roche) digestion following the manufacturer’s instructions. Afterwards, RNA was purified following standard procedures. RNA-seq analyses were performed on control leaves (0 mM NaCl) and on leaves of plants treated with 100 mM NaCl.

### LTR-REs and gene expression analyses

RNA-seq analyses were performed on control leaves (0 mM NaCl) and on leaves of plants treated with 100 mM NaCl at 24 and 48 days after the beginning of the experiment. Twelve cDNA libraries of control and stressed leaves of *F. carica*, obtained as described by Vangelisti et al. [33], were downloaded from NCBI SRA at BioProject accession code PRJNA508874.

The quality of each library was assessed by Bioanalyzer 2100 (Agilent Technologies, Santa Clara, CA, USA) and sequencing was performed by using Illumina HiSeq2000. The quality of the paired-end (125 bp length) read sequences was checked with FastQC (v. 0.11.5 [93] and trimmed with Trimmomatic, cropping the first 15 bases and last 10 bases and removing adapters.

cDNA reads were mapped to whole transcriptome and full-length LTR-REs by using the CLC Genomics Workbench with the same parameters described for DNA alignment. The expression level of LTR-REs was calculated as reads per kilo base per million mapped reads (RPKM, [94]); RPKM is a normalized value of expression which allows to compare transcription of sequences within and among libraries. RPKM is calculated by counting the reads aligned on a sequence on the total number of reads in a library, and on the sequence length [94]. Only LTR-REs with RPKM > 1 in at least one library were considered expressed. To detect possible differentially expressed LTR-REs between the control and salt stressed leaves during the two time points, a pairwise likelihood test on edgeR [95] was applied to read counts derived from alignment. A False Discovery Rate (FDR) [96] was used for the resulting *p*-values. We considered those LTR-REs that showed an absolute Fold-Change > 2 and FDR < 0.05 as differentially expressed.

To evaluate the expression pattern of genes close to LTR-REs, the area in the 50 kbp up- and down-stream of transposable elements was investigated. Genes in close proximity to these elements were extracted by using coordinates provided by the GFF file of the *F. carica* genome [1] and exploiting the intersect function of BEDtools v2.29.2 [97]. Expression values for genes in close proximity to LTR-REs were measured in terms of RPKM. These genes were split in classes by the range of RPKM. Then, expression was compared between genes lying in close proximity to expressed and not expressed LTR-REs. Differences were subjected to a Fisher’s exact test and considered significant for FDR < 0.05.

Functional analysis of genes was conducted by analysing GO terms provided with the *F. carica* gene annotation table [1]. The overall distribution of GO terms was visualised with WEGO 2.0 [98]. Blast2GO was used to perform enrichment analysis between the set of close genes to expressed LTR-REs and the whole transcriptome by using Fisher’s exact test [99]; GO terms were selected as significant for FDR corrected *p-*values < 0.05. Enriched GO terms were further summarised using REVIGO [100] by the tiny parameter option.

## Supplementary Information

**Additional file 1: Table S1.** Number of mapped read onto full-length LTR-REs for each library of cDNA of control (C) and salt treated (S) plants after 24 and 48 days of treatment.

**Additional file 2: Table S2.** Genes which showed a stable expression pattern in control (C) and stressed leaves (S) during 24 and 48 days. Only genes that showed a low/medium expression similar to LTR-REs are reported.

**Additional file 3: Figure S1.** Box plot of RPKM values of expression for LTR-REs in *F. carica* leaves. The four box plots represent the expression level in leaves of the control (C) and salt treated (S) plants after 24 and 48 days since the beginning of the experiment.

## Data Availability

DNA sequencing data have been deposited in the NCBI repository under BioProject accession code PRJNA565858. The fig genome has been deposited at DDBJ/ENA/GenBank under the accession VYVB00000000. The version described in this paper is version VYVB01000000. The collection of TEs is available at the repository sequence page of the Department of Agriculture, Food, and Environment of the University of Pisa (http://pgagl.agr.unipi.it/sequence-repository/). RNA sequencing data have been deposited in the NCBI repository under Bioproject accession code PRJNA508874. Intermediary files and other miscellaneous information are available from the corresponding author on reasonable request.

## Abbreviations

FDR: False Discovery Rate
LTR: Long Terminal Repeat
LTR-RE: Long Terminal Repeat-Retrotransposon
MY: Million Years
PacBio: Pacific Biosciences
RE: Retrotransposon
RPKM: Reads Per Kilo base per Million mapped reads
SMRT: Single-Molecule Real-Time
TGS: Third-Generation-Sequencing
