# Supplementary material for: LTR-retrotransposon dynamics in common fig (Ficus carica L.) genome
Source: BMC Plant Biol. 2021 May 17;21:221. doi: 10.1186/s12870-021-02991-x (PMC8127270; doi:10.1186/s12870-021-02991-x)
Supplement: Supplementary file 1 — Additional file 1: Table S1. Number of mapped read onto full-length LTR-REs for each library of cDNA of control (C) and salt treated (S) plants after 24 and 48 days of treatment. [file 12870_2021_2991_MOESM1_ESM.pdf]

Additional File 1

Table S1: Number of mapped read onto full-length LTR-REs for each library of cDNA of control (C) and salt treated (S) plants after 24 and 48 days of treatment

Libraries	Mapped reads per library	Percentage of mapped reads per library	Average mapped reads per library	Average % of mapped reads per library
**C24 (1)**	22,656	0.31		
**C24 (2)**	17,742	0.26	19,854	0.25
**C24 (3)**	19,164	0.19		
**S24 (1)**	22,494	0.28		
**S24 (2)**	22,454	0.31	21,077	0.28
**S24 (3)**	18,283	0.26		
**C48 (1)**	16,755	0.17		
**C48 (2)**	13,036	0.12	26,042	0.15
**C48 (3)**	48,335	0.16		
**S48 (1)**	17,311	0.22		
**S48 (2)**	15,778	0.21	16,515	0.20
**S48 (3)**	16,456	0.16		
