# Supplementary material for: LTR-retrotransposon dynamics in common fig (Ficus carica L.) genome
Source: BMC Plant Biol. 2021 May 17;21:221. doi: 10.1186/s12870-021-02991-x (PMC8127270; doi:10.1186/s12870-021-02991-x)
Supplement: Supplementary file 2 — Additional file 2: Table S2. Genes which showed a stable expression pattern in control (C) and stressed leaves (S) during 24 and 48 days. Only genes that showed a low/medium expression similar to LTR-REs are reported. [file 12870_2021_2991_MOESM2_ESM.pdf]

Vangelisti et al. - LTR-retrotransposon dynamics in common fig (*Ficus carica* L.) genome

Additional File 2

Table S2: Genes which showed a stable expression pattern in control (C) and stressed leaves (S) during 24 and 48 days. Only genes that showed a low/medium expression similar to LTR-REs are reported

Gene id	Gene description	C24 average RPKM	S24 average RPKM	C48 average RPKM	S48 average RPKM	
FCD_00014786	replication protein A 70 kDa DNA-binding subunit B	2.329	2.035	2.263	2.408	**Low expressed genes**
FCD_00014605	hydroxyproline O-galactosyltransferase GALT6	2.217	2.214	2.024	2.289	
FCD_00014836	muscle M-line assembly protein	2.333	1.847	1.960	2.367	
FCD_00012326	protein NETWORKED 1B	2.112	2.230	1.978	2.675	
FCD_00015515	receptor-like protein kinase	2.566	2.989	2.040	2.282	
FCD_00007704	pentatricopeptide repeat-containing protein	3.316	3.095	3.055	3.334	
FCD_00024994	histone-lysine N-methyltransferase setd3	2.531	2.067	1.887	2.492	
FCD_00022355	pentatricopeptide repeat-containing protein	2.432	2.807	1.966	1.823	
FCD_00035321	O-fucosyltransferase 30	2.531	2.664	1.845	2.109	
FCD_00032058	FYVE domain-containing protein/DUF500 domain-containing protein	2.205	2.705	2.476	2.565	
FCD_00002834	phosphatidylinositol 3-kinase	80.430	76.176	83.916	81.224	**Medium expressed genes**
FCD_00003684	serine/threonine-protein kinase DDB_G0282963	82.670	84.861	87.461	91.906	
FCD_00014654	DUF1664 domain-containing protein	78.598	79.636	88.421	89.491	
FCD_00004778	proline--tRNA ligase, cytoplasmic	84.429	83.860	89.268	97.744	
