# Supplementary material for: LTR-retrotransposon dynamics in common fig (Ficus carica L.) genome
Source: BMC Plant Biol. 2021 May 17;21:221. doi: 10.1186/s12870-021-02991-x (PMC8127270; doi:10.1186/s12870-021-02991-x)
Supplement: Supplementary file 3 — Additional file 3: Figure S1. Box plot of RPKM values of expression for LTR-REs in F. carica leaves. The four box plots represent the expression level in leaves of the control (C) and salt treated (S) plants after 24 and 48 days since the beginning of the experiment. [file 12870_2021_2991_MOESM3_ESM.pdf]

Vangelisti et al. - LTR-retrotransposon dynamics in common fig (*Ficus carica* L.) genome

Additional File 3

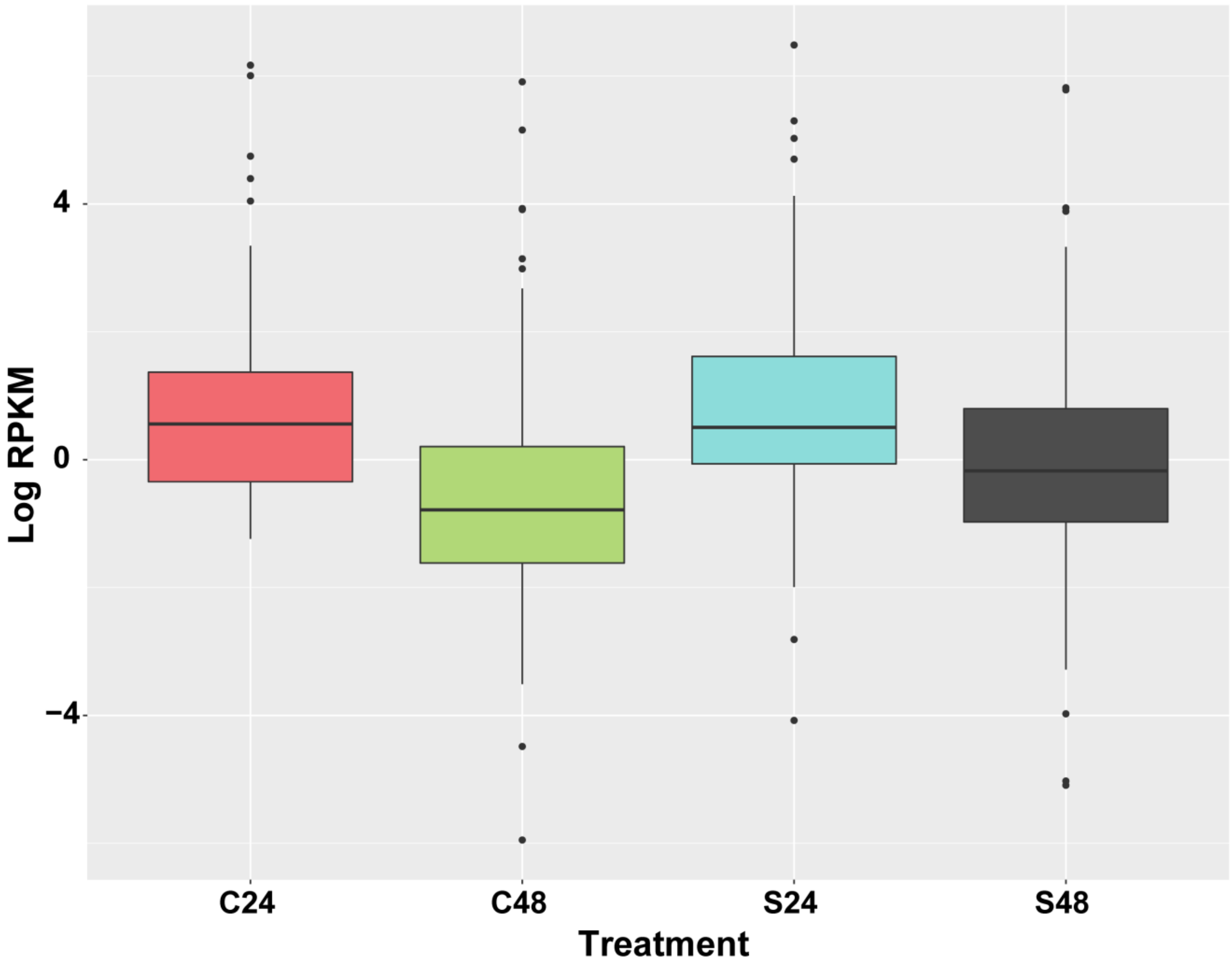

Figure S1: Box plot of RPKM values of expression for LTR-REs in *F. carica* leaves. The four box plots represent the expression level in leaves of control (C) and salt treated (S) plants after 24 and 48 days since the beginning of the experiment. The bars represent the mean of RPKM (in logarithmic scale) of all LTR-REs, the boxes include 75 % of LTR-REs
